# Prediction of all-cause mortality with malnutrition assessed by controlling nutritional status score in patients with heart failure: a systematic review and meta-analysis

**DOI:** 10.1017/S1368980021002470

**Published:** 2022-07

**Authors:** Huiyang Li, Peng Zhou, Yikai Zhao, Huaichun Ni, Xinping Luo, Jian Li

**Affiliations:** Department of Cardiology, Huashan Hospital, Fudan University, No.12, Mid Urumuqi Road, Jingan District, Shanghai 200040, People’s Republic of China

**Keywords:** Controlling nutritional status, Heart failure, All-cause mortality, Meta-analysis

## Abstract

**Objective:**

The aim of this meta-analysis was to investigate the association between malnutrition assessed by the controlling nutritional status (CONUT) score and all-cause mortality in patients with heart failure.

**Design:**

Systematic review and meta-analysis.

**Settings:**

A comprehensively literature search of PubMed and Embase databases was performed until 30 November 2020. Studies reporting the utility of CONUT score in prediction of all-cause mortality among patients with heart failure were eligible. Patients with a CONUT score ≥2 are grouped as malnourished. Predictive values of the CONUT score were summarized by pooling the multivariable-adjusted risk ratios (RR) with 95 % CI for the malnourished *v*. normal nutritional status or per point CONUT score increase.

**Participants:**

Ten studies involving 5196 patients with heart failure.

**Results:**

Malnourished patients with heart failure conferred a higher risk of all-cause mortality (RR 1·92; 95 % CI 1·58, 2·34) compared with the normal nutritional status. Subgroup analysis showed the malnourished patients with heart failure had an increased risk of in-hospital mortality (RR 1·78; 95 % CI 1·29, 2·46) and follow-up mortality (RR 2·01; 95 % CI 1·58, 2·57). Moreover, per point increase in CONUT score significantly increased 16% risk of all-cause mortality during the follow-up.

**Conclusions:**

Malnutrition defined by the CONUT score is an independent predictor of all-cause mortality in patients with heart failure. Assessment of nutritional status using CONUT score would be helpful for improving risk stratification of heart failure.

Heart failure remains a growing public health burden^(1)^. Despite advancements in medical care, heart failure is still the main cause of mortality, morbidity and hospital readmission^(2)^. Malnutrition is a common problem for heart failure^(3)^. Heart failure patients with malnutrition are associated with poor prognosis than those with normal nutrition^(4,5)^. Considering nutritional status can affect disease progression, early detection of malnutrition may improve risk classification of heart failure patients.

Controlling nutritional status (CONUT) score depending on the blood parameters of albumin, total cholesterol and lymphocyte counts is a simple screening tool for evaluation of nutritional status of inpatients^(6)^. According to the CONUT score, individuals with a CONUT score ≥2 are grouped into malnourished (mild 2–4; moderate, 5–8; and severe 9–12).Malnutrition assessed by the CONUT score was correlated with heart failure severity and rehospitalisation in acute heart failure^(7)^. The predictive utility of malnutrition defined by the CONUT score has attracted much attention in patients with heart failure^(8,9,10,11,12,13,14,15)^. However, there are conflicting results on the predictive value of CONUT score in these studies^(13,14,15)^.

No previous systematic review and meta-analysis has systematically addressed the predictive value of CONUT score in patients with heart failure. The aim of this systematic review and meta-analysis was to examine the association of malnutrition defined by the CONUT score with all-cause mortality in patients with heart failure.

## Methods

### Literature search

The current meta-analysis is reported according to the Preferred Reporting Items for Systematic Reviews and Meta-Analyses guidelines^(16)^. Two independent authors comprehensively searched the medical databases including PubMed and Embase until 30 November 2020. The following keywords in combination were applied for literature search: ‘Controlling nutritional status’ OR ‘CONUT’ AND ‘heart failure’ AND ‘mortality’ OR ‘death’ (see online Supplemental Text S1). Reference lists of relevant articles were manually scanned to identify any additional studies.

### Inclusion and exclusion criteria

Inclusion criteria were as follows: (1) population: patients with heart failure; (2) exposure: baseline CONUT score; (3) comparison: patients with malnutrition defined by the CONUT score ≥2 *v*. those with normal nutritional status; (4) outcome measures: all-cause mortality; (5) study design: prospective or retrospective observational studies and (6) reported multivariable-adjusted risk estimate of survival outcome for the malnourished *v*. normal nutritional status or per point CONUT score increase. The exclusion criteria included (1) studies did not select the normal nutrition (CONUT score 0–1) as reference; (2) outcome measures were not of interest and (3) lack of detailed risk summary for the outcome or reported unadjusted risk estimate.

### Data extraction and quality assessment

The following data of each study were abstracted surname of the first author, publication year, study design (retrospective or prospective), country, type of patients, sample size, percentage of men, mean/median age or age range, left ventricular ejection fraction (LVEF) at baseline, categorical or continuous analysis of CONUT score, most fully adjusted risk estimate, follow-up duration and adjusted variables. The Newcastle–Ottawa Scale was applied to assess the methodological quality of the included studies^(17)^. Studies with a total score with 7 points or higher were considered as high quality. Two independent authors conducted the data extraction and quality assessment. Any disagreements were settled through discussion.

### Data synthesis

The predictive value of CONUT score was expressed by pooling multivariable-adjusted risk ratios (RR) with 95 % CI for the CONUT score ≥2 *v*. CONUT score 0–1 or per point CONUT score increase. The *I* ^2^ statistics and Cochran’s *Q* test were applied to investigate the heterogeneity between studies. Value of *I* ^2^ statistics ≥50 % or *P* < 0·10 of the Cochran *Q* test revealed the presence of significant heterogeneity, and then a random effects model was selected. We selected a fixed-effect model in case of without significant heterogeneity. The robustness of the pooling risk summary was investigated by leave-out one study sensitivity analysis. Potential publication bias was checked by funnel plots if more than ten studies analyzed in the outcome. Subgroup analyses were conducted by the study design, patients’ number, mean/median age, follow-up duration, type of heart failure and baseline LVEF. Data analyses were conducted using Stata 12.0 (Stata Corp., College Station, TX).

## Results

### Search results and studies characteristics

**Fig. 1:**
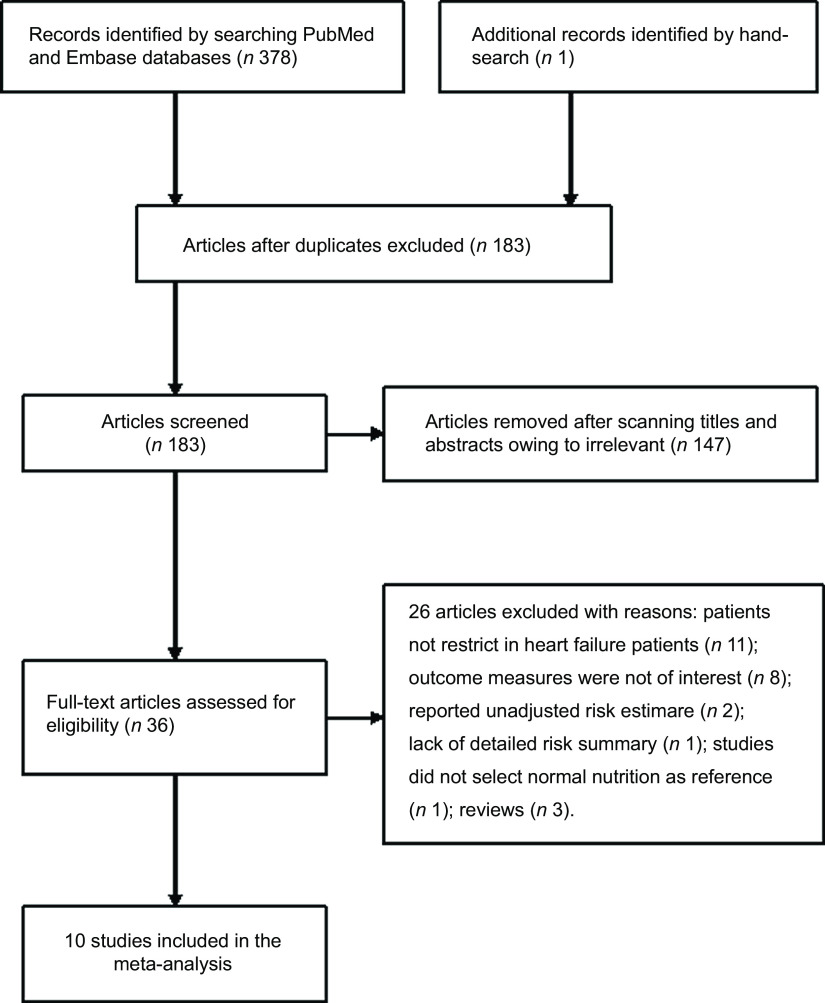
Flow chart showing studies selection process

A total of 183 potentially relevant articles were identified after the removal of duplications. Of which, 147 articles were excluded after scanning the titles and abstracts. Thirty-six articles were retrieved for full-text evaluation. After applying our pre-defined inclusion and exclusion criteria, twenty-six articles were further removed for different reasons (Fig. 1). Thus, ten studies^(8,9,10,11,13,14,18,19,20,21)^ were ultimately included in this meta-analysis.

The major characteristics of the included studies are summarised in Table 1. These studies were published from 2017 to 2020 and conducted in UK, Italy, Turkey, Japan, China and Taiwan. Seven studies^(8,9,10,11,13,14,20,21)^ were retrospective designs and two^(18,19)^ were prospective studies. The number of patients of the eligible studies ranged from 170 to 1120, with a total of 5196 heart failure patients. The mean/median age of the patients ranged from 61 to 80 years old. Follow-up duration was up to 3·4 years. Regarding the methodological quality, eight studies^(8,9,11,13,14,19,20,21)^ were grouped as high quality (see online Supplemental Table S1).

**Table 1:** Main characteristic of the included studies

Author/year	Region	Study design	Patients (% male)		Age (years)		LVEF (%)		Cut-off value of CONUT	Outcomes/RR	95 % CI	Follow-up (years)	Adjusted for variables
Iwakami 2017^(8)^	Japan	R	AHF 635	62	75	12	38	17	CONUT ≥ 2 Per point increase	Total death 2·01 1·23	1·08, 3·72* 1·08, 1·39	1·0	Age, sex, SBP, Hb, eGFR, serum sodium, BMI, statin use, history of malignancy, liver disease, reactive airway disease and depression
Sze 2017^(13)^	UK	R	HF 265	62	80	72–86	NP		Per point increase	Total death 1·06	0·89, 1·27	1·6	Age, sex, Hb, atrial fibrillation, NT-proBNP, creatinine, Na and presence of CAD
Nishi 2017^(9)^	Japan	R	HF 482	61·8	71·7	13·6	40·5	15·2	CONUT ≥ 2 Per point increase	Total death 2·38 1·14	1·39, 4·06 1·04, 1·25	1·5	Age, sex for categorical analysis; age, sex, BMI, history of HF hospitalisation, Hb, eGFR, BNP and therapeutic agents
La Rovere 2017^(10)^	Italy	R	HF 466	86	61	11	33·7	10·6	Per point increase	Total death 1·42	1·11, 1·81	1·0	Multivariable analysis
Shirakabe 2017^(14)^	Japan	R	AHF 458	66	76	67–82	40	28–53	CONUT ≥ 2	Total death 1·49	0·99, 2·22*	1·0	Multivariable analysis
Chien 2019^(11)^	Taiwan	R	AHF 1120	39·4	77·2	12·6	≥50		Per point increase	Total death 1·08	1·02, 1·13	3·4	Age, sex, BMI, SBP, heart rate, prior HF, hypertension, CVD, DM, atrial fibrillation, hyperlipidaemia, eGFR and BNP
Geng 2019^(18)^	China	P	AHF 505	64	72	14	NP		CONUT ≥ 2	Total death 1·77	1·20, 3·01	In-hospital	Multivariable analysis
Sze 2020^(19)^	UK	P	HF 467	67	76	69–82	45	35–54	CONUT ≥ 2 Per point increase	Total death 3·05 1·28	1·58, 5·85 1·13, 1·45	1·5	Age, BMI, cardiac rhythm, NYHA class, Charlson score, NT-proBNP, Hb and eGFR
Uemura 2020^(20)^	Japan	R	AHF 170	59·4	67·6	15·1	48	32–61	CONUT ≥ 2 Per unit increase	Total death 2·31 1·13	1·16, 4·58 1·00, 1·29	3·0	Age, sex, BMI, history of HF admission, CAD, Hb, eGFR and LVEF
Alatas 2020^(21)^	Turkey	R	AHF 628	53·7	74·7	11·8	NP		CONUT ≥ 5	Total death 1·79	1·13, 2·83	In-hospital	Age, sex, NT-proBNP and presence of CAD

LVEF, left ventricular ejection fraction; CONUT, controlling nutritional status; RR, risk ratio; R, retrospective; AHF, acute heart failure; SBP, systolic blood pressure; eGFR, estimated glomerular filtration rate; HF heart failure; NP, not provided; NT-proBNP, amino-terminal pro-brain natriuretic peptide; BNP, brain natriuretic peptide; DM, diabetes mellitus; P, prospective; NYHA, New York Heart Association; CAD, coronary artery disease.

* Results from pooling the CONUT score subgroup in a fixed-effect model.

### Categorical analysis of CONUT score on all-cause mortality

**Fig. 2:**
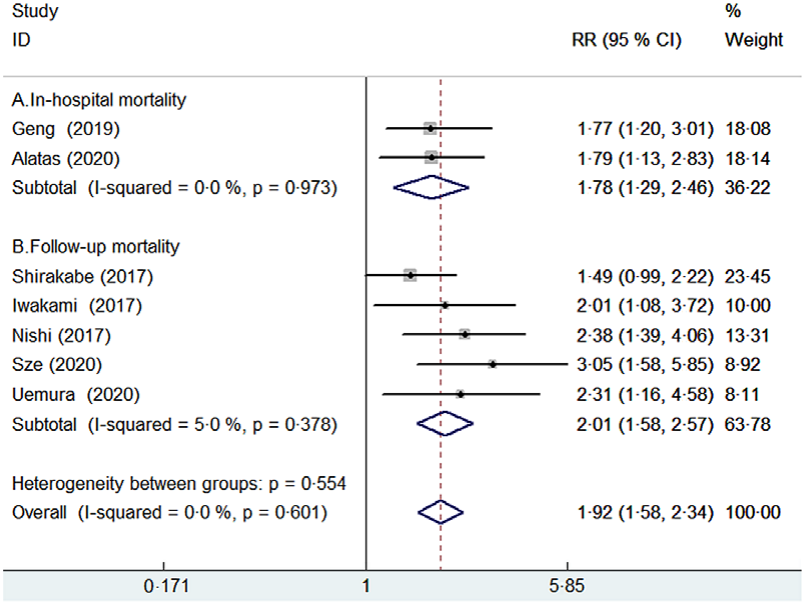
Forest plots showing pooled RR with 95% CI of all-cause mortality for patients with malnutrition *v*. those with normal nutritional status

Seven studies^(8,9,14,18,19,20,21)^ provided the categorical analysis of the CONUT score. A fixed-effect model meta-analysis indicated that the pooled RR of all-cause mortality was 1·92 (95 % CI 1·58, 2·34) for the CONUT score ≥2 *v*. CONUT score 0–1, without significant heterogeneity (*I* ^2^ = 0 %; *P* = 0·601; Fig. 2). Sensitivity analysis by excluding anyone study each time did not significantly alter the pooling risk summary (pooled RR varied from 1·84 to 2·08 and low 95 % CI varied from 1·50, 1·67). Subgroup analysis showed that malnutrition (CONUT score ≥2) was associated with an increased risk of in-hospital mortality (RR 1·78; 95 % CI 1·29, 2·46) and follow-up mortality (RR 2·01; 95 % CI 1·58, 2·57). In addition, significantly predictive values of malnutrition were consistently observed in each pre-defined subgroup (Table 2).

**Table 2:** Subgroup analyses on all-cause mortality by categorical analysis

Subgroup	Number. of studies	Pooled RR	95 % CI	Heterogeneity between studies
Study design				
Prospective	2	2·12	1·45, 3·09	*P* = 0·182; *I* ^2^ = 43·8 %
Retrospective	5	1·86	1·48, 2·34	*P* = 0·655; *I* ^2^ = 0·0 %
Sample sizes				
<500	4	1·83	1·37, 2·44	*P* = 0·943; *I* ^2^ = 0·0 %
≥500	3	2·01	1·54, 2·63	*P* = 0·240; *I* ^2^ = 28·7 %
Median/mean age				
<75 years	4	1·97	1·53, 2·55	*P* = 0·787; *I* ^2^ = 0·0 %
≥75 years	3	1·86	1·38, 2·51	*P* = 0·182; *I* ^2^ = 41·4 %
Type of HF				
AHF	5	1·76	1·41, 2·20	*P* = 0·837; *I* ^2^ = 0·0 %
All HF	2	2·63	1·74, 3·98	*P* = 0·566; *I* ^2^ = 0·0 %
Baseline LVEF				
<45 %	3	1·82	1·36, 2·42	*P* = 0·367; *I* ^2^ = 0·2 %
≥45 %	2	2·67	1·66, 4·29	*P* = 0·566; *I* ^2^ = 0·0 %
Follow-up duration				
≤1 year	4	1·71	1·35, 2·16	*P* = 0·857; *I* ^2^ = 0·0 %
>1 year	3	2·54	1·78, 3·62	*P* = 0·806; *I* ^2^ = 0·0 %

RR, risk ratio; HF heart failure; AHF, acute heart failure; LVEF, left ventricular ejection fraction.

### Continuous analysis of CONUT score on all-cause mortality

**Fig. 3:**
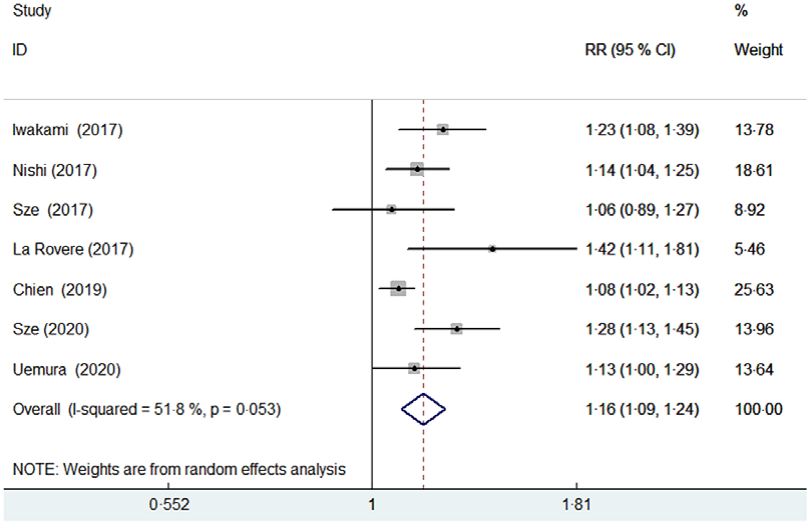
Forest plots showing pooled RR with 95% CI of all-cause mortality for per point increase in CONUT score

Seven studies^(8,9,10,11,13,19,20)^ reported the predictive value of the CONUT score by continuous analysis. A random effect model meta-analysis indicated that the pooled RR of all-cause mortality was 1·16 (95 % CI 1·09, 1·24) for per point CONUT score increase, with significant heterogeneity (*I* ^2^ = 51·8 %; *P* = 0·053; Fig. 3). Sensitivity analysis by removal of individual study each turn did not significantly change the pooling risk summary (pooled RR varied from 1·14 to 1·19 and low 95 % CI varied from 1·07, 1·11).

### Publication bias

Owing to less than recommended arbitrary number of ten studies, we did not construct the funnel plots or perform Begg’s and Egger’s test to check the publication bias^(22)^.

## Discussion

To the best of our knowledge, this is the first systematic review and meta-analysis to assess the association of malnutrition assessed by the CONUT score with all-cause mortality in heart failure patients. This meta-analysis demonstrated that malnutrition assessed by the CONUT score ≥2 was associated with higher risk of all-cause mortality. Heart failure patients with malnutrition had a 1·92-fold exaggerated risk of all-cause mortality. Moreover, when analysed the predictive role value of CONUT score as a continuous variable, per point increase in CONUT score was associated with 16 % exaggerated risk of all-cause mortality. The current meta-analysis confirms the available evidence for nutritional evaluation by the CONUT score in risk classification of patients with heart failure. Additionally, each category increase in CONUT score conferred 37 % higher risk of all-cause mortality in hospitalised patients with heart failure^(23)^.

Our subgroup analysis revealed that the value of malnutrition assessed by the CONUT score in predicting follow-up mortality was stronger than in-hospital death. This finding suggested that the impact of malnutrition on all-cause mortality appeared to strengthen with the lengthening of the follow-up. Furthermore, subgroup analysis by types of heart failure indicated that the predictive performance of CONUT score may be different in acute and chronic heart failure. According to the results of subgroup analysis, malnutrition assessed by the CONUT score was associated with 1·76-fold and 2·63-fold higher risk of all-cause mortality in acute heart failure and total heart failure. These findings indicated that the impact of malnutrition on all-cause mortality may be stronger in chronic heart failure. In addition, the baseline LVEF may also affect the predictive value of malnutrition assessed by the CONUT score in the stratified analysis. The impact of malnutrition on all-cause mortality appeared to be stronger in patients with preserved LVEF than those with reduced LVEF. However, it should be emphasised that the results of subgroup analysis should be interpreted with caution because of the limited number of studies analysed.

A number of simple nutritional screening tools, such as prognostic nutritional index, geriatric nutritional risk index and CONUT score, have been introduced in the assessment of nutritional status in the clinical practice^(24)^. However, which index is superior nutritional assessment method for estimating prognosis has yet been established in heart failure patients. Previous meta-analysis has showed that malnutrition assessed by the geriatric nutritional risk index (calculated by the albumin, body weight and height) was associated with higher risk of all-cause mortality (RR 2·11; 95 % CI 1·72, 2·58) in heart failure patients^(25)^. However, geriatric nutritional risk index was calculated by the albumin, body weight and height. Body weight of the heart failure patients can be affected by oedema and use of diuretics, which is potentially inaccurate in these patients. Another meta-analysis showed that Mini Nutritional Assessment score exhibited the best value in predicting risk of mortality (RR 4·32; 95 % CI 2·30, 8·11) in heart failure patients than other tools^(26)^. However, Mini Nutritional Assessment was difficult to apply in routine clinical setting because this screening tool required a multidimensional evaluation of psychological problems, residential status, mobility, body weight, diets and medications^(27)^.

The exact mechanisms of predictive values of CONUT score in heart failure remain unclear. CONUT score is derived from serum albumin, total cholesterol and lymphocyte count. The parameters of the CONUT score reflect protein and lipid metabolism as well as immune defences. Both hypoalbuminemia^(28)^ and low total cholesterol^(29)^ have been considered as a predictor for worse outcomes in patients with heart failure. Peripheral lymphocyte counts as surrogate biomarker of immune status could independently predict 1-year mortality in patients with acute heart failure^(30)^. Therefore, the combination of inflammatory and nutritional status can synergetic improve the predictive significance.

Although malnutrition assessed by the CONUT score increased all-cause mortality in patients with heart failure, the type of death is largely unclear. Conflicting findings have reported on the association of CONUT score with cardiovascular death^(8,9)^. There may be different mechanistic association between malnutrition and mortality in acute or chronic heart failure. Malnutrition was mainly correlated with reduced cardiac output and subsequent hypoperfusion-associated neurohormonal and inflammatory activity as well as anorexia/malabsorption in patients with acute heart failure^(8)^. While in patients with chronic heart failure, malnutrition was correlated with systemic inflammation, renal dysfunction, immunocompetence and anaemia^(31)^. Future well-designed studies are required to investigate whether there are different mechanistic relationships between nutrition and cardiovascular death or all-cause mortality.

Over 20 % of hospitalised patients with heart failure had moderate-to-severe malnutrition assessed by CONUT score, prognostic nutritional index and geriatric nutritional risk index^(23)^. Loss of appetite and malabsorption induced by heart failure can lead to malnutrition. Considering the higher prevalence and adverse impact of malnutrition on the prognosis, heart failure patients should be routinely assessed for nutritional status^(32)^. Our meta-analysis highlights the importance to evaluate the nutritional status using CONUT score in patients with heart failure. Heart failure patients with higher CONUT score should be accepted more closely monitoring and active nutritional interventions. On the other hand, a multicentre, randomised, controlled clinical trial supported the survival benefit of nutritional intervention in malnourished patients with heart failure^(33)^. Future well-designed clinical trials are warranted to evaluate whether nutritional interventions could potentially improve outcomes in heart failure patients with malnutrition.

This meta-analysis had several potential limitations. First, majority of the analysed studies were retrospective designs, and the inherent selection bias may be existed in these studies. Second, cholesterol level is affected by the use of statins therapy, which could have confounded the assessment of nutritional status using the CONUT score. Third, predictive value of different degree of malnutrition defined by the CONUT score was not evaluated in the current meta-analysis due to insufficient data. Fourth, lack of adjusting both measured and not measured residual confounding variables such as baseline LVEF, Hb, BMI or therapeutic agents may have led to over-estimate the predictive values of the CONUT score. Finally, potential publication bias may affect the pooling risk estimates. However, we did not test the publication bias because of less than recommended arbitrary number of studies analysed.

## Conclusion

This meta-analysis consolidates the current evidence that malnutrition assessed by the CONUT score was associated with higher risk of all-cause mortality in patients with heart failure. Assessment of nutritional status using CONUT score can improve risk classification of heart failure. More well-designed prospective studies with large sample sizes are required to demonstrate the current findings.

## Supplementary material

For supplementary material accompanying this paper visit https://doi.org/10.1017/S1368980021002470. click here to view supplementary material
