# Supplementary material for: Prediction of all-cause mortality with malnutrition assessed by controlling nutritional status score in patients with heart failure: a systematic review and meta-analysis
Source: Public Health Nutr. 2021 Jun 30;25(7):1799–806. doi: 10.1017/S1368980021002470 (PMC9991799; doi:10.1017/S1368980021002470)
Supplement: Supplementary file 1 [file S1368980021002470sup.zip › S1368980021002470sup001.doc]

**Supplemental Text S1 – Search strategy for this meta-analysis**

Medical Databases---PubMed and Embase

Limits: English and Chinese language, publications until November 30, 2020

\# 1 (“heart failure” [MeSH Terms])

\# 2 (“Controlling nutritional status” [MeSH Terms] OR “CONUT” [Free Terms])

\# 3 (“mortality” [Free terms] OR “death” [Free terms])

\# 4 (#1) AND (#2) AND (#3)

In addition, reference lists of the analyzed studies and pertinent articles were manually reviewed.
