# Supplementary material for: Prediction of all-cause mortality with malnutrition assessed by controlling nutritional status score in patients with heart failure: a systematic review and meta-analysis
Source: Public Health Nutr. 2021 Jun 30;25(7):1799–806. doi: 10.1017/S1368980021002470 (PMC9991799; doi:10.1017/S1368980021002470)
Supplement: Supplementary file 1 [file S1368980021002470sup.zip › S1368980021002470sup002.doc]

~~Supplemental Table S1 Methodological quality assessment based on the Newcastle-Ottawa Scale~~

| Author/Year | Representativeness of the exposed cohort | Selection of the non exposed cohort | Ascertainment of exposure | Demonstration that outcome was not present at study start | Comparability of cohorts on the basis of the design or analysis | Assessment of outcome | Enough follow-up periods (> 1 years) | Adequacy of follow-up of cohorts | Total scores |
| --- | --- | --- | --- | --- | --- | --- | --- | --- | --- |
| Iwakami 2017 ^(8)^ | ★ | ★ | ★ | ★ | ★ | ★ |  | ★ | 7 |
| Sze 2017 ^(13)^ |  | ★ | ★ | ★ | ★★ | ★ | ★ | ★ | 8 |
| Nishi 2017 ^(9)^ |  | ★ | ★ | ★ | ★★ | ★ | ★ | ★ | 8 |
| La Rovere 2017 ^(10)^ |  | ★ | ★ | ★ | ★ | ★ |  | ★ | 6 |
| Shirakabe 2017 ^(14)^ | ★ | ★ | ★ | ★ | ★ | ★ |  | ★ | 7 |
| Chien 2019 ^(11)^ | ★ | ★ | ★ | ★ | ★ | ★ | ★ | ★ | 8 |
| Geng 2019 ^(18)^ | ★ | ★ | ★ | ★ | ★ | ★ |  |  | 6 |
| Sze 2020 ^(19)^ |  | ★ | ★ | ★ | ★★ | ★ | ★ | ★ | 8 |
| Uemura 2020 ^(20)^ |  | ★ | ★ | ★ | ★ | ★ | ★ | ★ | 7 |
| Alatas 2020 ^(21)^ | ★ | ★ | ★ | ★ | ★ | ★ |  | ★ | 7 |
